# Identification of Functional Subclasses in the DJ-1 Superfamily Proteins

**DOI:** 10.1371/journal.pcbi.0030010

**Published:** 2007-01-26

**Authors:** Ying Wei, Dagmar Ringe, Mark A Wilson, Mary Jo Ondrechen

**Affiliations:** 1 Department of Chemistry and Chemical Biology, Northeastern University, Boston, Massachusetts, United States of America; 2 Institute for Complex Scientific Software, Northeastern University, Boston, Massachusetts, United States of America; 3 Department of Biochemistry, Brandeis University, Waltham, Massachusetts, United States of America; 4 Department of Chemistry, Brandeis University, Waltham, Massachusetts, United States of America; 5 Rosenstiel Basic Medical Sciences Research Center, Brandeis University, Waltham, Massachusetts, United States of America; Peking University, China

## Abstract

Genomics has posed the challenge of determination of protein function from sequence and/or 3-D structure. Functional assignment from sequence relationships can be misleading, and structural similarity does not necessarily imply functional similarity. Proteins in the DJ-1 family, many of which are of unknown function, are examples of proteins with both sequence and fold similarity that span multiple functional classes. THEMATICS (theoretical microscopic titration curves), an electrostatics-based computational approach to functional site prediction, is used to sort proteins in the DJ-1 family into different functional classes. Active site residues are predicted for the eight distinct DJ-1 proteins with available 3-D structures. Placement of the predicted residues onto a structural alignment for six of these proteins reveals three distinct types of active sites. Each type overlaps only partially with the others, with only one residue in common across all six sets of predicted residues. Human DJ-1 and YajL from Escherichia coli have very similar predicted active sites and belong to the same probable functional group. Protease I, a known cysteine protease from *Pyrococcus horikoshii,* and PfpI/YhbO from *E. coli,* a hypothetical protein of unknown function, belong to a separate class. THEMATICS predicts a set of residues that is typical of a cysteine protease for Protease I; the prediction for PfpI/YhbO bears some similarity. YDR533Cp from *Saccharomyces cerevisiae,* of unknown function, and the known chaperone Hsp31 from E. coli constitute a third group with nearly identical predicted active sites. While the first four proteins have predicted active sites at dimer interfaces, YDR533Cp and Hsp31 both have predicted sites contained within each subunit. Although YDR533Cp and Hsp31 form different dimers with different orientations between the subunits, the predicted active sites are superimposable within the monomer structures. Thus, the three predicted functional classes form four different types of quaternary structures. The computational prediction of the functional sites for protein structures of unknown function provides valuable clues for functional classification.

## Author Summary

Genome sequencing has led to the discovery of many new gene products, proteins. These discoveries hold tremendous potential for totally new approaches to the diagnosis and treatment of disease. To realize this potential, one important step is to understand the function of the thousands of proteins whose function is currently unknown. One of these proteins of unknown function is human DJ-1, a protein that appears to play a protective role against Parkinson and other neurodegenerative diseases. Here we present a computational approach to the classification by function of DJ-1 and its family members. Eight DJ-1 family members, all with similar 3-D structure, are analyzed. Three different probable functional classes emerge from this analysis on six of the family members, all with a simple calculation.

## Introduction

Structural biology in the post-genome era faces the challenge of determination of function from 3-D structure, the critical next step toward the realization of the promises of genomics. On the order of 10^3^ protein structures in the Protein Data Bank (PDB) are annotated as “hypothetical” or of “unknown function,” and this number is increasing dramatically as structural genomics initiatives deposit large numbers of structures in the PDB. Functional annotation is usually dependent on sequence similarity to identify proteins that are expected to be similar in structure and therefore may be similar in function. Even when sequence comparison fails to find a closely related protein, the overall structural fold still may be similar to one that is already known. Such structural relationships, however, still do not necessarily identify a functional relationship. The reason for the discrepancy is that currently there is not adequate understanding of the relationship between macromolecular structure and function for most proteins. Thus, structural similarity in many cases has proved to be a poor guide to function. Many proteins with similar and recognizable folds have completely different functions, even sometimes when there is sufficient sequence similarity to consider them “homologous.” The best examples of this principle are the enzymes having the TIM (triosephosphate isomerase) barrel fold. The types of reactions catalyzed by proteins having this fold are numerous and varied.

Conversely, two proteins may have completely different folds, but catalyze the same reaction, with the same residues and configurations in the active site. A good example is the set of pyridoxal phosphate–dependent transaminases of fold types I and II. These proteins catalyze the same reaction, with active sites that are practically identical, but the two folds are completely different.

In addition, the important residues in an enzyme active site may not be obvious. Many reactions in biology may be characterized by the steps required to bring about any chemical transformation. The catalytic entities involved in each step, such as acids or bases, can be inferred from the known chemistry. Residues that can play these roles are well-defined; however, it is not so easy to determine which particular residues in a given protein are actually playing these roles. Ideally, a structure with substrate bound would resolve the question, but such structures are rarely available for proteins of unknown function. Therefore, another method is needed to identify residues involved in catalysis and molecular recognition. In this paper we demonstrate how a computational predictive tool can aid in the identification of the functionally important residues in proteins of unknown function.

We have previously reported on THEMATICS (theoretical microscopic titration curves), a simple and fast computational tool for the prediction of catalytic and recognition sites in proteins that requires only the 3-D structure of the query protein as input [1–7]. THEMATICS is based on Poisson–Boltzmann calculations of the electrical potential for the protein structure, calculation of the theoretical titration curves (average charge as a function of pH) for all of the ionizable residues, and then statistical analysis of the computed titration curves to identify the ones that deviate the most from typical Henderson–Hasselbalch behavior. Clusters in coordinate space of two or more residues with deviant theoretical titration behavior are considered predictive and indeed predict localized interaction sites in proteins with high recall (91%) and high precision, as measured by the low filtration ratio (the fraction of ionizable residues selected), of about 8%. Here we report on how these predictive tools can be used to aid the experimental study of proteins of unknown function.

In the present paper we focus on a family of structurally similar proteins of biomedical importance that apparently have different biochemical functions, the DJ-1 superfamily. Human DJ-1 is a protein of unclear function that apparently plays a neuroprotective role and is involved in the cellular response to oxidative stress [8]. Mutations of DJ-1 have been associated with certain forms of early onset Parkinson disease, and DJ-1 has been independently identified as a ras-dependent oncogene. Members of the DJ-1 superfamily have been annotated as proteases because of similarity to a bacterial protease. However, recent experimental evidence suggests that DJ-1 and some other family members are not proteases. The purpose of the present paper is to sort these structurally similar proteins into functional classes, based on theoretical predictions of active site residues and the spatial arrangements of these residues. We compare THEMATICS predictions with the experimental evidence that is currently available and argue that these structurally similar proteins fall into at least three distinct functional classes.

The catalytic power of an enzyme relies not only on the nature of the residues that aid catalysis, but also on their position relative to the substrate. The method that identifies residues in the active site of a structure therefore also locates their relative positions and defines the type of chemistry that is possible, and potentially the substrate that can be recognized. Here we show that the arrangements in space of the residues predicted by our method form structural motifs from which one can deduce important clues about functionality. We illustrate the principle with a set of structurally similar proteins with different probable functions. Our predictions enable the similar structures to be sorted into distinct functional categories.

## Results

A search [9] for structures similar to DJ-1 was performed, and 11 structures with a Dali Z score of 15 or higher and an RMSD of 2.3 or less were chosen. The next closest proteins had significantly lower Z scores (7.6 or lower) and higher RMSD (3.0 or higher). The structures included in the analysis are now described. Unlike some other members of the DJ-1 superfamily (PfpI family), human DJ-1 does not exhibit any significant protease activity. Another family member, the YajL (formerly labeled ThiJ) protein from *E. coli,* is of unknown function [10]. Protease I from P. horikoshii is a known cysteine protease [11], from which many other proteins in this group have been annotated in sequence databases. PfpI/YhbO from E. coli is a hypothetical protein of unknown function. YDR533Cp from S. cerevisiae is of unknown function [12]. The chaperone Hsp31 from E. coli is a known chaperone with some reported peptidase activity [13]. APC35852 from Bacillus stearothermophilus is a structural genomics protein of unknown function. Two E. coli structures with PDB IDs 1VHQ and 1OY1 are of the identical protein, with the sequences differing only at the C-terminal His tag. Both of these are structural genomics proteins, and the structures were determined by two different groups. 1VHQ is annotated as an enhancing lycopene biosynthesis protein, and 1OY1 is annotated as a putative sigma cross-reacting protein. All of these proteins are members of the DJ-1 superfamily and share closely related 3-D structures in their core fold. These 3-D structures are distinguished from one another by variable insertions into the core fold and by different quaternary structures. Table 1 summarizes the annotations for these proteins given in the databases of Pfam (http://www.sanger.ac.uk/Software/Pfam), Gene Ontology (http://www.geneontology.org/index.shtml), and the PDB (http://www.rcsb.org/pdb/home/home.do).

**Table 1:**
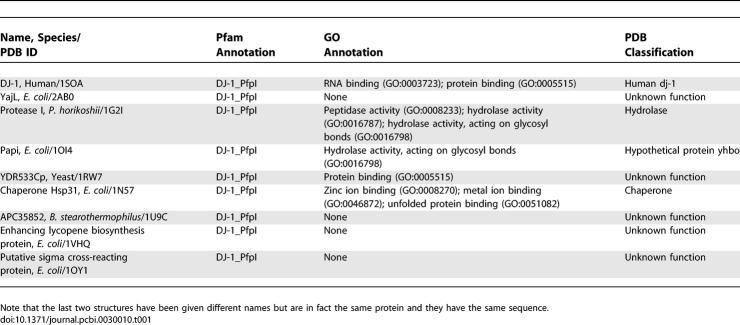
Annotations for the DJ-1 Superfamily Members, as Given by Pfam, GO, and the PDB

Two additional structures, Catalase I from Neurospora crassa and Catalase II from *E. Coli,* both have a domain of similar structure to DJ-1, but the catalytic sites are located in a different domain. For the two catalases, THEMATICS correctly predicts the catalytic sites and predicts nothing in the domains with structural similarity to DJ-1. There is no experimental evidence of any functional activity in the DJ-1 domain of these catalases. Therefore, these two catalases are excluded from the present analysis of functional classification of the DJ-1 superfamily members.

**Figure 1:**
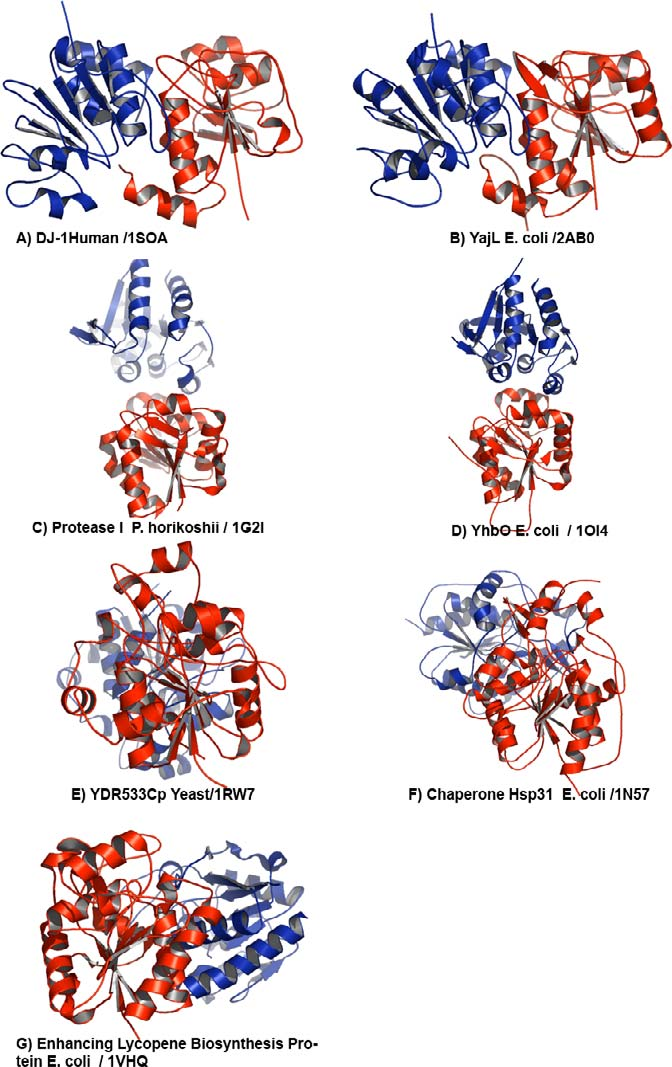
Dimer Structures for Seven DJ-1 Family Members with the Two Subunits Shown in Red and Blue (A) Human DJ-1; (B) YajL from *E. coli;* (C) Protease I from *P. horikoshii;* (D) YhbO from *E. coli;* (E) YDR533Cp from yeast; (F) Chaperone Hsp31 from *E. coli;* (G) the structural genomics putative Enhancing lycopene biosynthesis protein from E. coli. For all structures, the red subunits are oriented so that they are superimposable on each other. The relative positions of the blue subunits then illustrate the different types of dimer formation.

The different types of quaternary structures in the DJ-1 family are illustrated in Figure 1, showing ribbon diagrams of the dimer structures of the first six of the above DJ-1 family members plus the putative enhancing lycopene biosynthesis protein, with the two subunits colored red and blue in each structure. For all of the structures, the red subunits are oriented so that they are superimposable on each other without rotation. DJ-1 and YajL form similar dimer structures. Protease I and YhbO likewise are similar to each other, with dimer interfaces at a surface different from that of DJ-1/YajL. On the other hand, YDR533Cp and Hsp31 form quaternary structures that are different from each other, with the blue subunit attaching at a common face on the red subunit but at different orientation.

The DJ-1 family proteins illustrate the difficulty of functional annotation from sequence [14]. The sequence alignments for this set of proteins (ranging from 16%–35% identity) might mislead one into concluding that their functions are similar. Especially the presence of a cysteine in similar positions within each sequence was considered highly suggestive of function. Thus, originally DJ-1 was presumed to be a cysteine protease because of its sequence similarity to the known protease. Later Bandyopadhyay and Cookson [14] studied 311 sequence homologues and analyzed their alignments and phylogenetic trees. These authors report that this set of sequences may be sorted into distinct subgroups; proteins with similar annotations appear to cluster together into distinct clades. The subgroup closest to that of human DJ-1 is the bacterial YajL/ThiJ group, suggesting that DJ-1 may have evolved from bacterial thiamine synthesis genes that have assumed some other function in eukaryotes.

We have analyzed the DJ-1 sequence using the Joined Assembly of Function Annotations (JAFA) server (http://jafa.burnham.org) [15]. JAFA attempts to find consensus among five different sequence-based function annotation methods: GOFigure [16], GOblet [17], InterProScan [18], GOtcha [19], and PhydBac [20]. For human DJ-1, three of the five servers were unable to annotate the sequence and returned no predictions. GOFigure reported possible thiamin pyridinylase activity and possible peptidase activity, with the higher score given to the former annotation. The PhydBac analysis gave the highest score to iron ion binding, the next-highest score to heme binding, and the third highest to catalase activity; it also indicated possible biological roles in response to oxidative stress and in response to biotic stimulus. No consensus could be found among the five methods, and thus this sequence analysis is inconclusive.

A structural alignment of the monomers of the first six proteins indicates clearly that there are differences in residue arrangements that the sequence alignment cannot reveal. The residues identified as functionally important by THEMATICS are a subset of the structurally aligned residues. These predicted residues show spatial patterns that allow the different proteins to be sorted into groups. THEMATICS predictions, expressed as 3-D constellations of potentially important residues, strongly suggest probable functional groupings. Table 2 shows the THEMATICS predicted clusters for six proteins in the DJ-1 structural family. Structurally aligned residues are aligned in columns in Table 2. When the conserved cysteine residue is not predicted by THEMATICS, it is shown in Table 2 in parentheses.

**Table 2:**
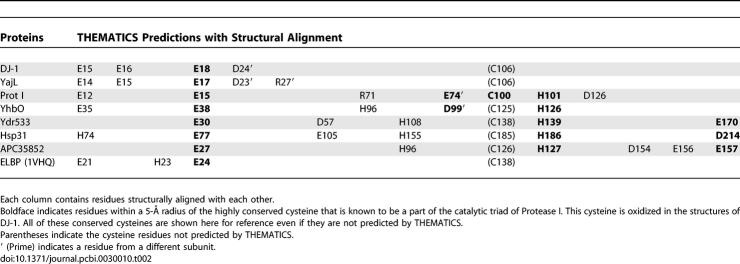
Structurally Aligned THEMATICS Predictions for Six Proteins

Note that one residue in a structurally conserved position is predicted to be important for all six proteins; this is a glutamate corresponding to the active site E15 of Protease I. Table 2 suggests that there are three different types of functional sites for the first six proteins.

For Protease I from *P. horikoshii,* THEMATICS predicts a cluster at the protease active site that includes the catalytic triad members C100, H101, and E74′; this triad is characteristic of cysteine proteases. Note that the prime indicates a residue from another subunit. A site similar but not identical to that of Protease I is predicted for PfpI/YhbO. Protease I and PfpI/YhbO have similar quaternary structures and similar interfaces. Their THEMATICS predicted sites are located at the interface.

For human DJ-1, THEMATICS finds a distinctly different cluster consisting of E15, E16, E18 and D24′, located adjacent to, but not coinciding with, the corresponding triad site. The sites predicted for DJ-1 and YajL are very similar. The predicted sites consist of four structurally aligned acidic residues. There is one residue difference between the two predictions, in that for YajL R27′ is also predicted. Again, the quaternary structures are similar to each other with similar interfaces.

For yeast YDR533Cp, THEMATICS predicts E30, D57, H108, H139, and E170, a cluster that overlaps with the corresponding triad site and also contains some additional residues that are not selected either for Protease I or for DJ-1. H139 is located in a position corresponding to that of the catalytic His101 of the Protease I triad, whereas E30 in the predicted YDR533Cp cluster is structurally aligned with E18 of human DJ-1. H108 and E170 in the predicted YDR533Cp cluster are not predicted for DJ-1 or Protease I, but the corresponding residues are predicted for the chaperone Hsp31. For YDR533Cp and Hsp31, the predicted sites are each contained within a given monomer. Indeed, the similarities in the predicted sites are apparent for the structurally aligned monomers of YDR533Cp and Hsp31, but these two proteins form dimers with different orientations between the subunits.

**Figure 2:**
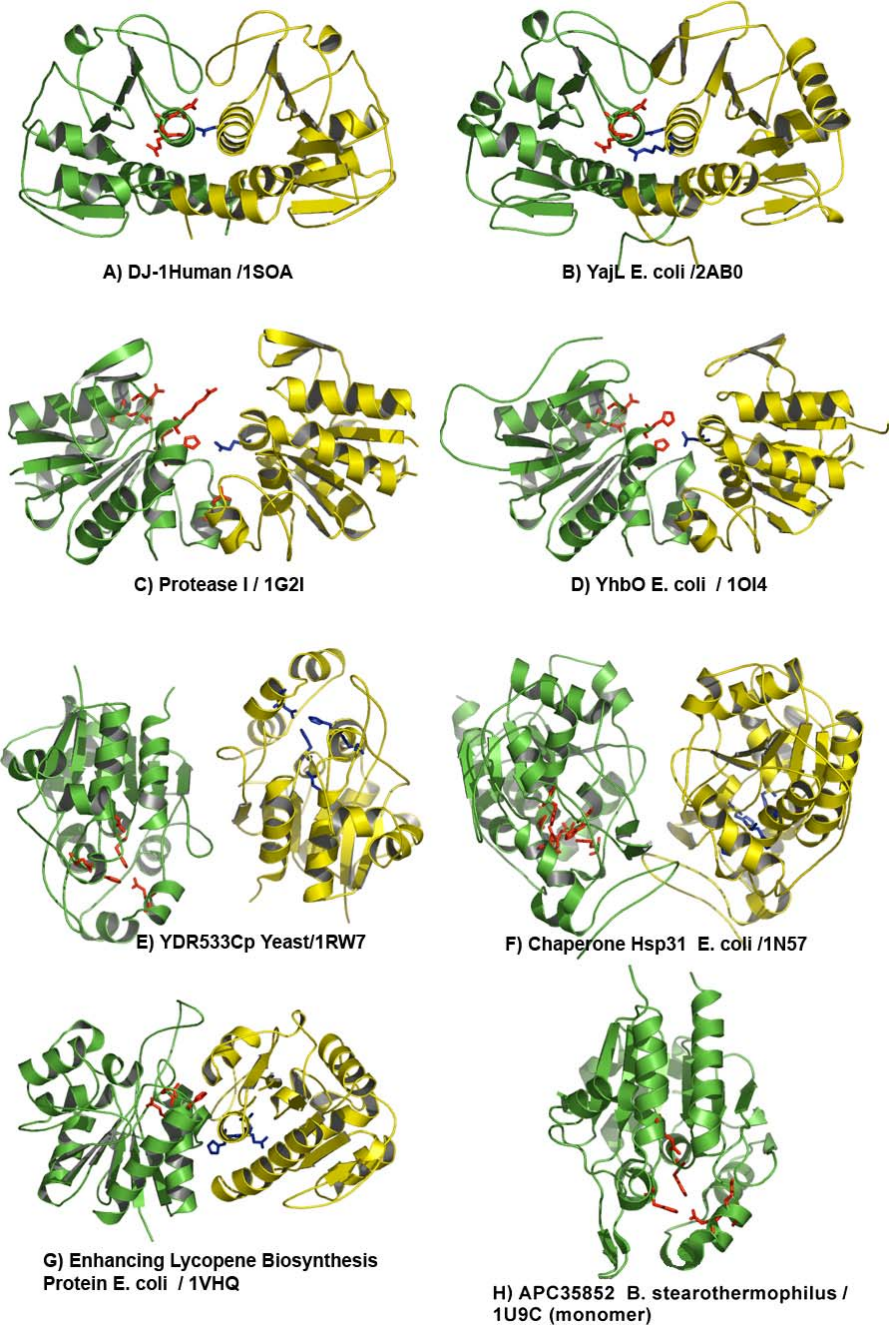
Ribbon Diagrams of Eight DJ-1 Family Proteins with Predicted Active Sites (A) Human DJ-1; (B) YajL *E. coli;* (C) Protease I *P. horikoshii;* (D) YhbO *E. coli;* (E) YDR533Cp yeast; (F) Chaperone Hsp31 *E. coli;* (G) putative Enhancing lycopene biosynthesis protein *E. coli;* (H) APC35852 B. stearothermophilus. The subunit backbones are shown in yellow and green. Residues predicted by THEMATICS to be active site residues are shown in red (from the green subunit) and blue (from the yellow subunit).

Figure 2 shows a side-by-side comparison of the predicted active site residues in the dimer structures of Human DJ-1 (Figure 2A) and YajL (Figure 2B), Protease I (Figure 2C) and PfpI/YhbO (Figure 2D), and YDR533Cp (Figure 2E) and Hsp31 chaperone (Figure 2F), plus the prediction for the dimer structure of Enhancing lycopene biosynthesis protein (1VHQ; Figure 2G) and for the monomer structure of APC35852 (1U9C, Figure 2H). Ribbon diagrams are shown with the backbone of the “a” subunit of the dimer in green and the side chains of the THEMATICS predicted residues from the “a” chain in red; the backbone of the “b” subunit is shown in yellow with the side chains of the THEMATICS predictions from the “b” chain in blue. Note the similar spatial arrangements and locations of the predicted sites for DJ-1 and YajL. Predictions for Protease I and PfpI/YhbO are also similar in spatial arrangement in their relative positions in the structures. YDR533Cp and Hsp31 have predicted clusters located within each subunit, removed from the dimer interface, unlike the first four structures. Note also that the way in which the monomers of YDR533Cp and of Hsp31 come together to form the dimer is different, although the monomers and the predicted sites within them are similar. The two structural genomics protein structures 1VHQ (Figure 2G) and 1U9C (Figure 2H) have predicted sites quite different from those of the first three pairs of structures.

**Figure 3:**
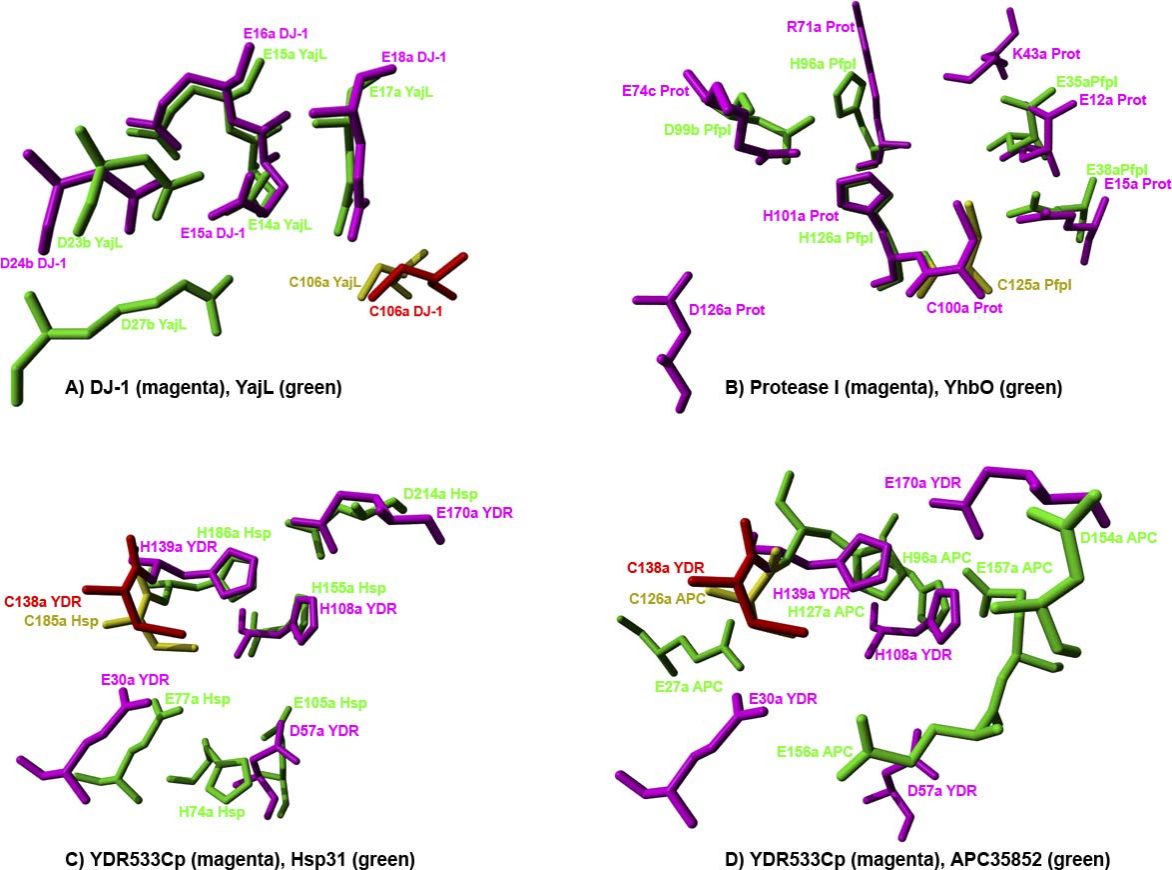
Superpositions of the THEMATICS Predicted Active Site Residues (in Green and Magenta) for DJ-1 Family Members (A) DJ-1 (magenta), YajL (green); (B) Protease I (magenta), YhbO (green); (C) Ydr533 (magenta), Hsp31 (green); (D) Ydr533 (magenta), APC35852 (green). The conserved cysteine residues that are not THEMATICS-positive residues are included for comparison purposes and are shown in yellow and red.

Figure 3 shows superpositions of the THEMATICS-predicted active site residues in magenta and green. Note the similarities in the predicted sites for Figure 3A, DJ-1 (magenta) and YajL (green); Figure 3B, Protease I (magenta) and YhbO (green); and Figure 3C, YDR533Cp (magenta) and Hsp31 (green). The yellow and red residues are conserved cysteine residues that are not THEMATICS positives. They are shown in the picture for comparison purposes. This conserved cysteine is shown in Figure 3A, YajL (yellow) and DJ-1 (red); Figure 3B, YhbO (yellow); Figure 3C, Hsp31 (yellow) and YDR533Cp (red); and Figure 3D, APC35852 (yellow) and YDR533Cp (red). The conserved cysteine in Protease I is a THEMATICS positive residue and is shown in Figure 3B in magenta. Even though YDR533Cp and Hsp31 have different quaternary structures, their THEMATICS-predicted active sites are the same except that Hsp31 has one additional histidine residue, H74. Superposition of their monomers yields nearly identical active site predictions for the remaining five residues. Figure 3D shows a superposition of the predicted residues of APC35852 with those of YDR533Cp.

While the analysis illustrated in Figure 3 suggests three different functional classes for those six structures with a common fold, there are probably additional functions for this 3-D structure. For instance, one domain of Catalase-1 (PDB ID 1SY7) [21] is structurally aligned with DJ-1; its catalase active site is in a different domain and is correctly predicted by THEMATICS; nothing is predicted in its DJ-1 domain, consistent with available experimental information. The structural genomics protein 1VHQ, annotated as Enhancing Lycopene Biosynthesis Protein, has a predicted site that somewhat resembles that of DJ-1 but is not clearly coincident with any of the structures studied. The structural genomics protein APC35852 (PDB ID 1U9C) is a monomeric protein, and THEMATICS predicts the site [E27, H96, H127, D154, E156, E157]. This prediction is closest to those for YDR533Cp and Hsp31. The E27 is structurally aligned with the glutamate that is common to the THEMATICS predictions for the structures of all of the first six proteins, the H96 and H127 are structurally aligned with two predicted histidines in YDR533Cp (H108 and H139), as shown in Figure 3D, and in Hsp31 (H155 and H186), while the E156 and E157 are not structurally aligned with any of the predicted residues for the above six proteins.

## Discussion

It has been shown previously that a relatively small group (of about five to seven members) of functionally important residues constitutes a 3-D signature that can be used to identify proteins in a superfamily [22]. Given that the different functional classes within the superfamily have evolved to affect different chemical transformations and to recognize different substrate molecules, it is likely that the full list of residues involved in catalysis and/or in recognition in each structure will contain not just signature residues of the superfamily but also residues characteristic of the particular functional class within the superfamily. THEMATICS is designed to identify exactly those characteristic residues involved in catalytic activity and in substrate specificity [1,2,4,5].

The predicted THEMATICS spatial clusters for the selected members of the DJ-1 family enable us to sort them into groups with similar predicted active sites and hence presumably similar function. In particular, the spatial arrangements of the THEMATICS predicted residues for DJ-1 and YajL are similar and form one such group. The predictions for these two structures are different by one residue, R27′, but this residue is close to the threshold between positive (predicted) and negative (not predicted). The difference between the two predicted sites is small enough to indicate a likely common function for the two structures.

Predictions for Protease I and PfpI/YhbO form similar, but not identical, spatial motifs and may constitute a distinct probable functional class. While the present analysis suggests that Protease I is the closest functional relative of YhbO, the two predicted sites do show some differences, and therefore one cannot conclude that YhbO is a cysteine protease. Indeed, Abdallah et al. recently reported [23] that YhbO exhibits neither protease nor chaperone activity.

Ydr533c and the chaperone Hsp31 form yet a third probable functional class. The predicted sites for these two latter proteins are contained within each subunit, and the two proteins exhibit different quaternary structures. Thus, in spite of sequence similarity, it is likely that these six proteins belong to at least three different functional classes.

Note that the six proteins have similar primary, secondary, and tertiary structures, yet the three predicted functional classes have different quaternary structures and different predicted functional sites. The three predicted functional classes are consistent with the positions of these proteins in the cladogram of Bandyopadhyay and Cookson [14]. The phylogenetic tree and the present method provide very different but complementary types of information. The cladogram indicates which proteins are the closest neighbors in the evolutionary history, based on sequence, while the present method identifies important functional residues and active site structural motifs, based on the 3-D structure. For the DJ-1 superfamily, the two methods support similar conclusions about the likely functional subclasses.

Recently we have shown [7] that THEMATICS can make correct site predictions for comparative model structures. The question then arises, can the present method be used to annotate the members of the superfamily whose structures are not known? This depends on the quality of the model structures and is the subject of further investigation.

The facile identification of binding and recognition sites in proteins with a simple calculation provides important and time-saving clues in the determination of a protein's function.

## Materials and Methods

THEMATICS analysis was performed on the protein structures according to the procedures described by Ko et al. [1], using a Z score cutoff value of 0.99 in the statistical analysis and using a distance cutoff of 9.0 Å to form the clusters. Structural alignments were performed using a Combinatorial Extension method and the 3-D Protein Structure Comparison and Alignment Server (http://cl.sdsc.edu) [24]. Structures were rendered using the graphical programs PyMol (http://www.pymol.org) and Yasara (http://www.yasara.org/index.html).

## Supporting Information

### Accession Numbers

The accession numbers from the Protein Data Bank (http://www.rcsb.org/pdb/home/home.do) used in this paper are: human DJ-1 (1SOA), Catalase-1 (1SY7), Protease I from P. horikoshii (1G2I), PfpI/YhbO from E. coli (1OI4), YDR533Cp from S. cerevisiae (1RW7), chaperone Hsp31 from E. coli (1N57), Catalase II from *E. Coli* (1GGE), YajL (formerly labeled ThiJ) protein from E. coli (2AB0)*,* Enhancing lycopene biosynthesis protein (1VHQ), putative sigma cross-reacting protein (1OY1), and structural genomics protein APC35852 (1U9C).

## Abbreviations

PDB: Protein Data Bank
THEMATICS: theoretical microscopic titration curves
